# Association of Lower Extremity Arterial Calcification with Amputation and Mortality in Patients with Symptomatic Peripheral Artery Disease

**DOI:** 10.1371/journal.pone.0090201

**Published:** 2014-02-26

**Authors:** Chi-Lun Huang, I-Hui Wu, Yen-Wen Wu, Juey-Jen Hwang, Shoei-Shen Wang, Wen-Jone Chen, Wen-Jeng Lee, Wei-Shiung Yang

**Affiliations:** 1 Department of Internal Medicine, Taoyuan General Hospital, Taoyuan City, Taiwan; 2 Department of Internal Medicine, National Taiwan University Hospital, Taipei City, Taiwan; 3 Graduate Institute of Clinical Medicine, College of Medicine, National Taiwan University, Taipei City, Taiwan; 4 Department of Surgery, National Taiwan University Hospital, Taipei City, Taiwan; 5 Department of Nuclear Medicine, National Taiwan University Hospital, Taipei City, Taiwan; 6 Department of Nuclear Medicine and Cardiovascular Medical Center (Cardiology), Far Eastern Memorial Hospital, New Taipei City, Taiwan; 7 National Yang-Ming University School of Medicine, Taipei City, Taiwan; 8 Department of Emergency Medicine, Lotung Poh-Ai Hospital, Yilan County, Taiwan; 9 Department of Medical Imaging, National Taiwan University Hospital, Taipei City, Taiwan; Medical University of Graz, Austria

## Abstract

**Objective:**

The clinical implication of the coronary artery calcium score (CS) is well demonstrated. However, little is known about the association between lower extremity arterial calcification and clinical outcomes.

**Methods and Results:**

Eighty-two patients with symptomatic peripheral artery disease (age 61.0±12.4 years) were followed for 21±11 months. CSs, ranging from the common iliac artery bifurcation to the ankle area, were analyzed through noncontrast multidetector computed tomography images retrospectively. The primary endpoints of this study were amputation and mortality. Old age, diabetes, hyperlipidemia, and end-stage renal disease were associated with higher CSs. Patients with more advanced Fontaine stages also tended to have significantly higher CSs (*p* = 0.03). During the follow-up period (21±11 months), 29 (35%) patients underwent amputation, and 24 (29%) patients died. Among the patients who underwent amputation, there were no significant differences in CSs between the amputated legs and the non-amputated legs. In the Cox proportional hazard model with CS divided into quartiles, patients with CS in the highest quartile had a 2.88-fold (95% confidence interval [CI] 1.18–12.72, *p = *0.03) and a 5.16-fold (95% CI 1.13–21.61, *p* = 0.04) higher risk for amputation and all-cause mortality, respectively, than those with CS in the lowest quartile. These predictive effects remained after conventional risk factor adjustment.

**Conclusion:**

Lower extremity arterial CSs are associated with disease severity and outcomes, including amputation and all-cause mortality, in patients with symptomatic peripheral artery disease. However, the independent predictive value needs further investigation in large scale, prospective studies.

## Introduction

Arterial calcification can occur systemically in nearly all vascular beds, in both the medial and intimal layers, and are associated with atherosclerosis and arteriosclerosis. The prevalence of arterial calcification increases with age and is stimulated by several common cardiovascular risk factors. The clinical consequences depend on the underlying disease state and location of the calcification. Calcification in coronary arteries is comprehensively studied, in part because of its association with cardiovascular events, but also because of the computed tomography (CT)-based imaging modality. In the general population, the presence of coronary artery calcification increases cardiovascular risk above that predicted by traditional Framingham risk factors, suggesting the presence of nontraditional risk factors [1]. Both plain radiographs and ultrasound can detect vascular calcification. However, only CT-based methods, which allow for quantification, are used in clinical practice. Calcification in different locations, including the carotid artery, thoracic aorta, abdominal aorta, and renal artery, has been examined in a limited number of studies as well [2]–[5]. The associations between arterial calcification and mortality differed by vascular bed, suggesting that the location and severity of calcification in different vascular beds provides unique information for mortality [6].

Diabetes and end-stage renal disease (ESRD) are two important determinants of vascular calcification. Lower extremity arterial calcifications on multidetector computed tomography (MDCT) were analyzed in these patient populations, and calcium scores (CSs) were higher in patients with peripheral artery disease (PAD) and positively correlated with disease severity, determined by Fontaine stage [7], [8]. However, the CS data from patients with PAD without diabetes and ESRD and the association with outcome are lacking. The aim of our study was to evaluate the association of lower extremity CS with other cardiovascular risk factors and its roles in outcome prediction in patients with symptomatic PAD.

## Materials and Methods

### Ethics Statement

The study was approved by the institutional review board of National Taiwan University Hospital. Medical records and patient information were anonymized and de-identified prior to analysis.

### Study Design

This was a retrospective, observational, single-center study conducted at the National Taiwan University Hospital. The local ethics committee approved the protocol. A total of 144 patients with symptomatic lower extremity PAD (from 2007 to 2011, Fontaine stage II – IV) with MDCT available were surveyed initially. All of them had significant arterial stenosis (>50%) and/or thrombosis on lower extremity MDCT. Individuals with a history of lower extremity amputation, subjects whose MDCT scans did not contain noncontrast images, scans with extensive metal artifact or missed arterial segments were excluded. Finally, 82 patients with acceptable lower extremity MDCT images were included in the analysis. The follow-up period began on the date of MDCT evaluation and ended on Dec 31, 2012. Amputation and mortality data were obtained from medical records.

All demographic information about clinical symptoms, Fontaine stages, age, gender, hyperlipidemia, hypertension, diabetes mellitus, and comorbid diseases, including coronary artery disease (CAD) and cerebral vascular accident (CVA), was collected. Hypertension was defined as systolic blood pressure ≥140 mmHg, diastolic blood pressure ≥90 mmHg, and/or reported use of antihypertensive medication. Diabetes mellitus was defined as current use of antiglycemic medications or a random blood glucose level >200 mg/dL. Laboratory parameters, which were collected from medical records within 3-month before MDCT, included fasting serum levels of calcium, phosphate, glucose, hemoglobin A1c, uric acid, total cholesterol, triglycerides, and low-density lipoprotein cholesterol.

### Calcification Scoring of Lower Extremity Arteries

Patients underwent noncontrast-enhanced MDCT scanning of the lower extremity arteries with a 64-row MDCT scanner. Scans were performed using helical acquisition with kV = 120, mAs = 200, and with a field of view of 350 to 380 mm. From the acquired raw data, the scan was reconstructed in 5-mm thick slices. The average number of slices of lower extremities was approximately 210.

The scoring of calcification started at the junction of descending aorta and common iliac artery, and ended at the ankle. The calcium score (CS) of lower extremity was composed of 3 segments: the iliac-femoral (IF) segment, the above-knee (AK) segment, and the below-knee (BK) segment. The IF segment included common iliac, external iliac, internal iliac, and femoral arteries. The AK segment included superficial femoral, deep femoral, and popliteal arteries. The BK segment included anterior tibial, posterior tibial, and peroneal arteries. The CS was analyzed using standardized calcium scoring software (Extended Brilliance Workspace, Philips Medical Systems, Cleveland) by investigators who were blinded to the results of the patients’ clinical assessment and Fontaine’s severity categories. On cross-sectional images through the lower extremities, area of calcification with a cross-sectional area >1 mm^2^ and a density of >130 Hounsfield units were identified and scored. The CS for each segments of interest was determined and expressed as Agatston score according to the method described by Agatston et al [9].

### Statistical Analysis

Continuous variables were reported as mean ± standard deviation values, whereas categorical variables were reported as numbers and percentages. Group comparisons were performed using 2-sample *t* test for normally distributed variables and Wilcoxon rank-sum test for non-normally distributed variables. CSs which were not normally distributed were logarithmically transformed before further analysis. Univariable relationships between CSs and clinical variables were assessed with the Pearson correlation coefficient (*r*). Multiple stepwise regression analysis was performed to determine the independent parameters correlated with CSs. Event-free survival in high and low CS groups was illustrated by Kaplan-Meier curves and compared using the log-rank test. Cox proportional hazards models were used to evaluate risk factor adjusted associations of variables of interest with amputation and all-cause mortality. The results were presented as hazard ratios (HRs) and 95% confidence intervals (CIs). Analyses were performed by using the Stata statistical software (release 10.0, StataCorp LP, Texas, USA). All statistical tests were 2-sided, with *p*<0.05 considered statistically significant.

## Results

**Figure 1:**
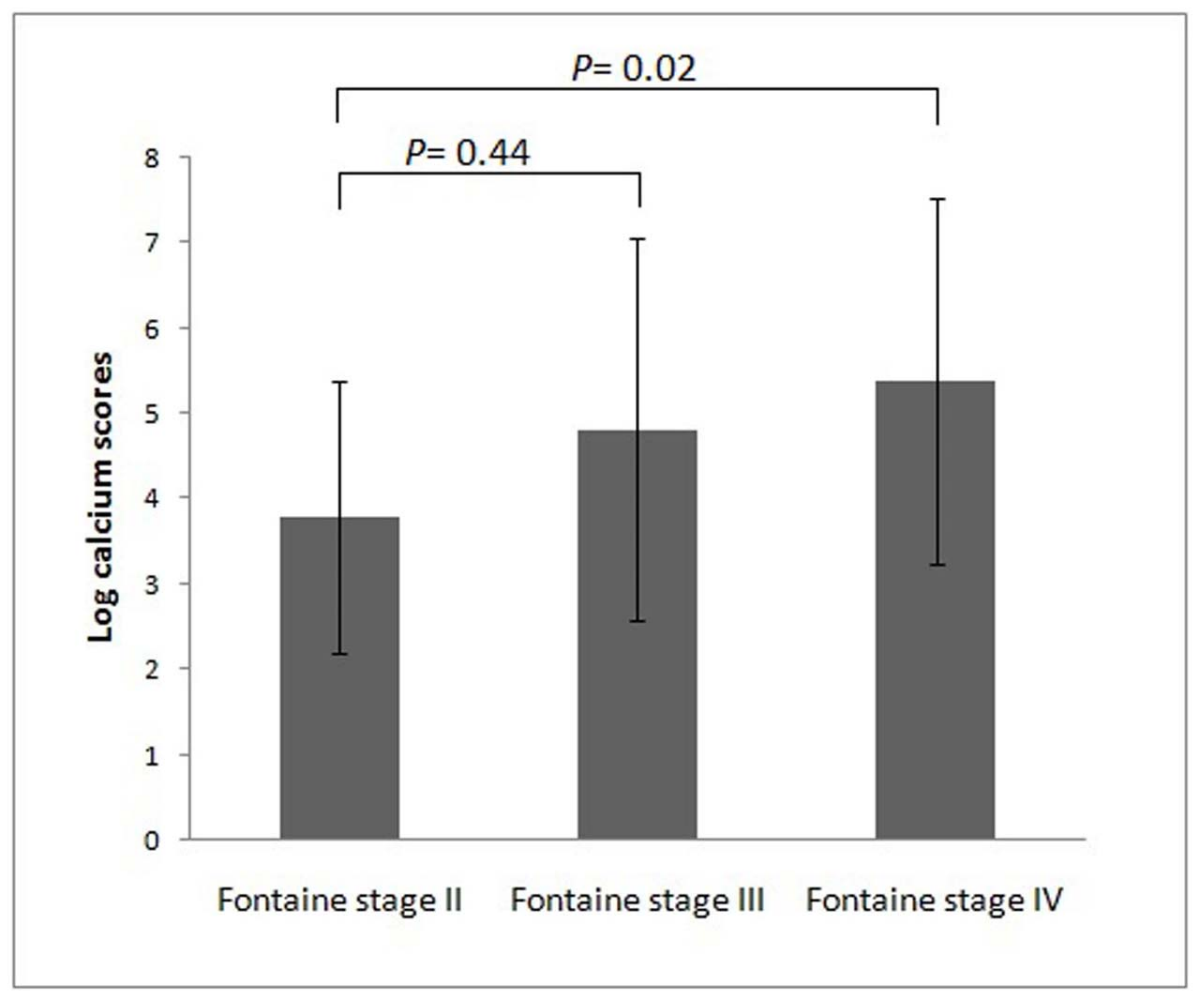
The lower extremity arterial calcium scores in peripheral artery disease patients with different Fontaine stages.

Among the 82 patients with symptomatic PAD, 68% of them were men, and the mean age was 69.5±12.1 years (Table 1). Of the total, 17 patients were classified into Fontaine stage II, 18 into stage III, and 47 into stage IV. The prevalence of hypertension, diabetes, and hyperlipidemia was extremely high in this cohort. Eighteen patients (22%) underwent regular hemodialysis, and 37 patients (45%) with chronic kidney disease (CKD) stage III – V. Subjects were further divided into high and low CS groups by using the cutoff level of 10942, the median CS of our population. The high CS group patients were older, with a higher prevalence of diabetes, hyperlipidemia, and ESRD. The pulse pressure, another indicator of arterial stiffness, was also wider in high CS group patients (mean 70 vs 61 mmHg, *p* = 0.04). However, higher CSs were not associated with a previous history of CAD and CVA. On the other hand, patients with PAD and more advanced Fontaine stages tended to have significantly higher CSs (*p* = 0.03, Figure 1). The correlations of CSs between different arterial segments, including IF, AK, and BK segments, are shown in Table 2. The total CS was highly correlated between the right and left legs (*r* = 0.98), and the segmental CSs were highly correlated between different arterial segments as well. The total CS was positively correlated with age and total cholesterol level (*r* = 0.30 and 0.29, respectively). To determine which variables were independently associated with CS, multiple stepwise regression analysis was performed and revealed that age (b = 0.05, *p* = 0.006), diabetes (b = 0.91, *p* = 0.048), and total cholesterol level (b = 0.01, *p* = 0.005) were independent predictors of the CS. We analyzed the association of the severity of arterial stenosis and CS in the AK segment. Among 54 patients who had unilateral severe stenosis in the superficial femoral artery, the CS was not higher in the ipsilateral segment (ipsilateral vs. contralateral: log CS, 3.84±2.21 vs. 3.77±2.42, *p* = 0.73).

**Table 1:** Baseline characteristics of study population.

Parameter	All patients	High calcium score	Low calcium score	*p* value
	N = 82	N = 41	N = 41	
Age (yr)	69.5±12.1	72.4±10.6	66.6±12.9	0.03
Male gender	56 (68%)	28 (68%)	28 (68%)	1
BMI (kg/m^2^)	23.9±4.2	22.8±3.5	24.9±4.5	0.04
Hypertension	63 (77%)	32 (78%)	31 (76%)	0.79
Diabetes mellitus	50 (61%)	30 (73%)	20 (49%)	0.02
Hyperlipidemia	51 (62%)	30 (73%)	21 (51%)	0.04
ESRD	18 (22%)	15 (37%)	3 (7%)	0.001
Smoker	30 (37%)	17 (41%)	13 (32%)	0.36
CAD history	27 (33%)	13 (32%)	14 (37%)	0.48
CVA history	16 (20%)	8 (20%)	8 (20%)	1
Systolic BP (mmHg)	141±20	145±19	137±20	0.16
Pulse pressure (mmHg)	65±16	70±15	61±17	0.04
Medications				
ACEi/ARBs	32 (39%)	20 (49%)	12 (29%)	0.07
Beta-blockers	39 (48%)	20 (49%)	19 (46%)	0.83
Statins	17 (21%)	9 (22%)	8 (20%)	0.79
Cilostazol	36 (44%)	19 (46%)	17 (41%)	0.66
Creatinine (mg/dl)*	1.2 (0.9, 1.9)	1.3 (0.95, 4.4)	1.1 (0.89, 1.3)	0.01
Total cholesterol (mg/dl)*	173 (143, 205)	186 (156, 208)	162 (134, 184)	0.04
Triglyceride (mg/dl)*	137 (98, 209)	156 (98, 202)	119 (93, 223)	0.60
LDL-C (mg/dl)	99±30	104±32	96±29	0.35
Uric acid (mg/dl)	6.27±2.15	6.21±2.34	6.34±1.98	0.84
Fasting glucose (mg/dl)*	107 (90, 137)	108 (87, 201)	104 (93, 125)	0.53
Hemoglobin A1c (%)	7.1±1.7	7.2±1.6	7.0±1.7	0.69
Calcium (mg/dl)	2.29±0.20	2.30±0.26	2.28±0.12	0.70
Phosphate (mg/dl)	4.4±1.6	4.8±1.6	3.9±1.6	0.33
Calcium scores*	10943 (2176, 36774)	36773 (18903, 59704)	2176 (976, 6535)	<0.0001

* Presented as median (25^th^, 75^th^ percentile) and analyzed by Wilcoxon rank-sum test.

ACEi/ARB: angiotensin converting enzyme inhibitor/angiotensin II receptor blocker; BMI: body-mass index; CAD: coronary artery disease; CVA: cerebral vascular accident; ESRD: end-stage renal disease; LDL-C: low-density lipoprotein cholesterol.

**Table 2:** Correlation between segmental calcium scores (CS) and biochemical parameters.

	r	*p* value
CS (left vs. right)	0.98	<0.0001
IFCS vs. AKCS	0.80	<0.0001
IFCS vs. BKCS	0.73	<0.0001
AKCS vs. BKCS	0.88	<0.0001
CS vs. age	0.30	0.007
CS vs. body-mass index	−0.08	0.52
CS vs. systolic BP	0.06	0.66
CS vs. creatinine	0.26	0.06
CS vs. total cholesterol	0.29	0.02
CS vs. triglyceride	0.22	0.07
CS vs. LDL-C	0.16	0.24
CS vs. fasting glucose	0.20	0.15

Calcium score (CS) was logarithmically transformed before analysis.

AKCS: above-knee calcium score; BKCS: below-knee calcium score; IFCS: iliac-femoral calcium score; LDL-C: low-density lipoprotein cholesterol.

**Figure 2:**
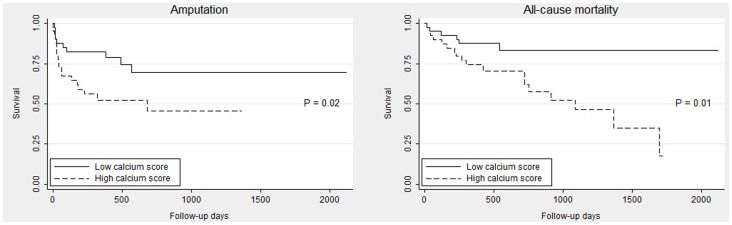
Kaplan-Meier survival curves for amputation and all-cause mortality according to lower extremity arterial calcium scores with the median as cutoff value in patients with symptomatic peripheral artery disease.

During the follow-up period (21±11 months), 29 (35%) patients received lower extremity amputations, and 24 (29%) patients died. The cause of death was critical limb ischemia-related sepsis in 10 patients, myocardial infarction in 5 patients, pneumonia in 3 patients, gastrointestinal bleeding in 2 patients, and malignancy in 2 patients, and others in 2 patients. Figure 2 shows the Kaplan-Meier survival curves for amputation and all-cause mortality. Patients with PAD in the high CS group had significantly higher amputation rates and mortality rates than those in the low CS group. Among the 29 patients who underwent amputation, there was no significant difference in baseline CS between the amputated legs and the non-amputated legs (Log CS, 5.0±2.0 vs. 4.9±2.0, *p* = 0.88). In the Cox proportional hazard model with CS in quartiles, patients with CS in the highest quartile had a 2.88-fold (95% confidence interval [CI] 1.18–12.72, *p = *0.03) and a 5.16-fold (95% CI 1.13–21.61, *p* = 0.04) higher risk for amputation and all-cause mortality, respectively, than those with CS in the lowest quartile. After traditional cardiovascular risk adjustment, patients with high CS were still associated with higher risk for amputation and mortality (*p* for trend: 0.01 and 0.046, Table 3). In segmental CS analyses, the CSs in all three segments were still indicators for lower extremity amputation after multivariate adjustment, but the predictive effects were significantly attenuated for all-cause mortality.

**Table 3:** Multivariate Cox proportional hazards analyses of calcium scores for lower extremity amputation and all-cause mortality.

Parameter	All calcium score		IFCS		AKCS			BKCS	
	HR (95% CI)	*p* value	HR (95% CI)	*p* value	HR (95% CI)	*p* value		HR (95% CI)	*p* value
**Amputation**									
Quartile 1	Reference		Reference		Reference			Reference	
Quartile 2	4.32 (0.73–25.49)	0.11	1.54 (0.31–7.73)	0.60	24.37 (1.82–326.09)	0.02		1.36 (0.28–6.62)	0.71
Quartile 3	4.69 (0.67–32.55)	0.12	5.31 (0.87–32.31)	0.07	83.09 (4.40–1568.6)	0.003		1.21 (0.09–4.24)	0.61
Quartile 4	12.25 (1.75–85.61)	0.01	7.29 (1.30–40.75)	0.02	39.09 (2.16–707.15)	0.01		8.04 (1.18–54.95)	0.03
* p* for trend		0.01		0.008		0.04			0.04
**All-cause mortality**									
Quartile 1	Reference		Reference		Reference			Reference	
Quartile 2	5.23 (0.43–63.11)	0.19	1.51 (0.20–11.30)	0.69	3.70 (0.30–45.04)		0.31	1.29 (0.20–8.47)	0.79
Quartile 3	14.36 (1.24–164.60)	0.03	2.68 (0.39–18.58)	0.32	9.89 (0.81–120.29)		0.07	2.30 (0.33–16.27)	0.40
Quartile 4	10.29 (0.85–124.71)	0.07	5.81 (0.84–39.97)	0.07	10.32 (0.85–125.33)		0.07	2.18 (0.25–18.90)	0.48
* p* for trend		0.046		0.03			0.05		0.38

Adjusted for age, gender, diabetes, hypertension, smoking, total cholesterol and eGFR.

AKCS: above-knee calcium score; BKCS: below-knee calcium score; IFCS: iliac-femoral calcium score.

## Discussion

To the best of our knowledge, this is the first published study which comprehensively, from pelvis to ankle and from trunk to major branches, evaluated lower extremity arterial calcification on MDCT in a symptomatic PAD population. Our study demonstrated that lower extremity arterial CS, which was higher in patients with old age, diabetes, hyperlipidemia, and ESRD, was associated with amputation and all-cause mortality in patients with symptomatic PAD.

Vascular calcification is associated with most conventional cardiovascular risk factors, including dyslipidemia, diabetes, obesity, and hypertension [10]. Calcium depositions in arterial walls increase with age and are reported in nearly 30% of Americans aged ≥45 years [11]. Among our patients with PAD, only 2 of them, age 40 and 52 years, did not have calcium deposition in their lower extremity arteries. The multivariate regression analysis also demonstrated that age, diabetes, and hyperlipidemia were the independent determinants of lower extremity CS. The CSs were significantly higher in our PAD patients with ESRD than those without (ESRD vs. non-ESRD: log CS, 5.72±2.22 vs. 4.20±2.07, *p* = 0.02). Similar results were also demonstrated in other studies. However, even in the 64 non-ESRD patients, high CSs were still associated with a 3.27-fold risk of all-cause mortality (95% CI 1.12–9.60, *p* = 0.03) than those with low CSs.

Calcification in the cardiovascular system can be classified into intimal atherosclerotic calcification, medial arterial calcification, and cardiac valve calcification [12]. The intimal and medial calcification could not be differentiated on the non-contrast MDCT images. However, the medial calcification, but not the intimal atherosclerotic calcification, might be the major contributor of arterial calcification in lower extremity PAD [13]. Intimal calcification, which is associated with atherosclerosis, is characterized by lipid accumulation, inflammation, fibrosis, and development of focal plaques. Medial calcification, also referred as Monckeberg’s sclerosis, directly increases arterial stiffness and is widespread in persons with metabolic disorders such as diabetes and ESRD. Clinical consequences of calcification include heart failure, valvular sclerosis and stenosis, ventricular hypertrophy, diastolic dysfunction, and hypertension [14]. Experimental and clinical studies have shown that arterial calcification is an active and complex process in which the vascular smooth muscle cells are involved and synthesize a group of bone-associated proteins, including alkaline phosphatase, osteocalcin, osteopontin, and collagen-rich extracellular matrix [15], [16]. Other regulatory factors include bone morphogenetic proteins, receptor activator of nuclear factor-kB ligand, tumor necrosis factor-alpha, fetuin-A, oxidative stress, hyperphosphatemia, and vitamin D [12]. Vascular calcification is recognized as a significant, independent predictor for cardiovascular events. A recent meta-analysis of 30 prospective cohort studies demonstrated the consistent finding that the presence of calcification poses an increased risk for cardiovascular and all-cause mortality [17]. In peripheral arteries, it independently predicts amputation and mortality in ESRD [18]. In our study, we extended the conclusion to all subjects with symptomatic PAD.

Although CS was associated with future amputation risk, data from coronary artery calcification studies demonstrated that CS was less correlated with the degree of stenosis in the same individual [19]. Our findings in lower extremity arteries also demonstrated that there was no correlation between the severity of segmental arterial stenosis and CS. This also explains why higher lower extremity CS did not predict ipsilateral leg amputation in individual patients with PAD.

Calculating the overall CS from the iliac to ankle is extremely time-consuming. Since CS from different segments was highly correlated, analysis of segmental CS might be more practical in clinical evaluation. Our results demonstrated that all three segmental CSs were independent predictors for lower extremity amputation. To determine the risk of amputation, analyzing only one segmental CS seems to be acceptable. However, none of these segmental CSs were associated with overall mortality. Guzman et al reported an increased amputation risk in 118 patients with PAD and high tibial artery calcification score [20]. A study conducted by Ohtake et al also showed that BK segment CS was a major associating factor for critical limb ischemia in patients undergoing hemodialysis [7]. Because of the relative small sample size and event number, the clinical value of different segmental CSs needs to be further investigated in a large scale study.

Several drugs used to treat cardiovascular problems (statins, angiotensin converting enzyme inhibitors [ACEi], warfarin) may have effects on bone tissue metabolism. In our study population, the CSs were significantly higher in patients with ACEi/angiotensin II receptor blocker treatment than those without (log CS, 5.50±2.14 vs. 4.15±1.85, *p* = 0.009). Patients undergoing statins treatment also tended to have higher CSs (log CS, 5.46±2.30 vs. 4.49±1.97, *p* = 0.12). These results might reflect only the underlying disease associated with PAD and not the effect of medications on CS. Indeed, some studies have indicated the effectiveness of statins in restricting the progression of calcium accumulation in vessel walls [21], [22], but a more recent randomized clinical trial failed to prove this [23].

The gender difference in CS was not detected in our present study (men vs. women: log CS, 4.77±2.14 vs. 5.27±2.11, *p* = 0.33). Previous studies demonstrated that female sex hormones, which increase bone density and inhibit osteoclast activity, play an important role in bone tissue metabolism [24]. Postmenopausal women with high serum estradiol levels had reduced coronary CSs independent of age and other coronary risk factors, and estrogen therapy also reduced the vascular calcification [25], [26]. Because all of our female patients were postmenopausal, it might explain the relatively high CS among them.

Our present study had some limitations. First, the small sample size, which leaded to wide confidence interval, was the major limitation of current study. However, if we took CS as continuous variable into regression model, the CS was still associated with amputation outcome with narrow confidence interval (1.000002–1.000004) and significant *p* value (*p* = 0.03). Besides, the Kaplan-Meier survival curves for both outcomes were also widely separated. Therefore, we believed that the significant association between CS and outcomes could not be neglected, although the risk ratio might not be precisely estimated. Second, the soft tissue calcification, especially in patients with ESRD, might interfere with our interpretation of arterial calcification on MDCT. Third, our study population was a group of patients with advanced PAD; more than half of the patients were Fontaine stage IV. We need to be careful to extend the conclusion to non-selective PAD patients. Finally, no blood samples were collected at the time of CT scan. We therefore could not analyze several important circulating mineralization factors in our current study.

## Conclusions

In our systemic PAD patients, lower extremity arterial calcification was symmetric distributed and associated with the Fontaine stages. Moreover, high CS in lower extremity arteries predicted worse outcomes, including amputation and overall mortality. The role of lower extremity arterial calcification deserves further comprehensive investigation, and CS should be taken into consideration as a novel parameter upon conventional risk factors in all patients with PAD.
